# Hodgkin Lymphoma in a Thymic Cyst: Report of a Case with Multiple Secondary Neoplasms

**DOI:** 10.1155/2010/795037

**Published:** 2010-06-02

**Authors:** Saeeda Almarzooqi, Sue Hammond, Samir B. Kahwash

**Affiliations:** ^1^Nationwide Children's Hospital, J 0336, Anatomic Pathology, 700 Children's Drive, Columbus, OH 43205, USA; ^2^Department of Pathology and Laboratory Medicine, Nationwide Children's Hospital, 700 Children's Drive, Columbus, OH 43205, USA; ^3^The Ohio State University, Columbus, OH 43210, USA

## Abstract

The presentation of Hodgkin Lymphoma in a thymic cyst is rare. We describe a case in a 9 year-old boy, with a long follow-up course, complicated by two secondary neoplasms and a post bone marrow transplant lymphoproliferative disorder. We also review the literature on such presentations and second malignant neoplasms in childhood.

## 1. Introduction

Mediastinal disease in the context of Hodgkin lymphoma is a frequent finding. However, the association of a Hodgkin lymphoma with a thymic cyst is rarely reported [1]. Here, we describe a case in a 9-year-old boy, with a long follow-up course, complicated by two secondary neoplasms and a post bone marrow transplant lymphoproliferative disorder.

## 2. Case Report

**Figure 1:**
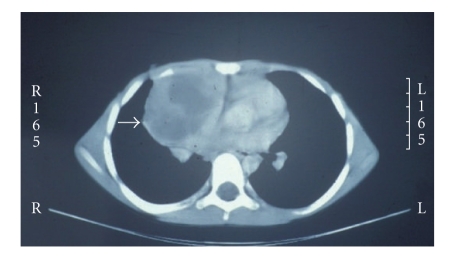
CT scan demonstrating a large cystic anterior mediastinal mass (arrow).

**Figure 2:**
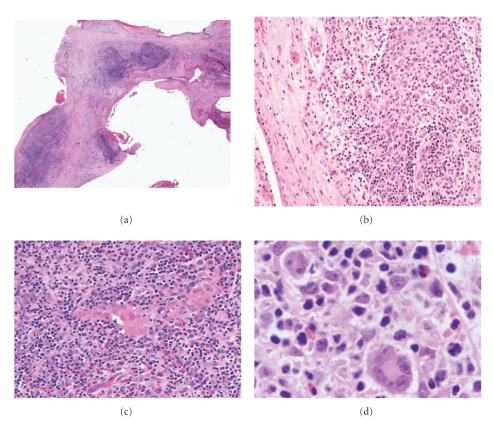
Hodgkin lymphoma in thymic cyst. (a) Wall of thymic cyst with nodules of cellular/inflammatory infiltrates (H&E. 25×). (b) Thymic cyst wall devoid of epithelium demonstrating the mixed inflammatory infiltrate in wall (H&E. 100×). (c) Hassel corpuscles surrounded by classic Reed-Sternberg cells in a mixed inflammatory background (H&E. 200×). (d) Classic Reed Sternberg cells present in a mixed inflammatory background of lymphocytes, histiocytes, and eosinophils (H&E. 400×).

A 9-year-old boy presented in 1993 with a one-week history of cough and fever. He had a WBC 16 000 K/cu mm, Hgb 12.7 g/dL. Chest X-ray (CXR) revealed a consolidated right middle lobe. Suspecting pneumonia, he received a course of antibiotics. His symptoms improved slightly but his CXR remained unchanged after the course of antibiotics. CT scan demonstrated mediastinal lymphadenopathy and a 13 cm anterior mediastinal mass (Figure 1). A needle aspirate of the intralesional fluid demonstrated a fibrinous exudate with benign mononuclear cells and epithelial lining cells consistent with a thymic cyst contents. Biopsy of the mass showed a mixed inflammatory cell infiltrate with atypical large mononuclear cells consistent with Reed-Sternberg cells. The neoplastic cells were present in nodules separated by dense fibrous septae (Figure 2). They showed positivity for CD15 with negative CD45 by immunoperoxidase method. CD30 was positive in the cytoplasm of Reed-Sternberg cells with perinuclear accentuation in some of the cells. A diagnosis of Hodgkin lymphoma, nodular sclerosis arising in conjunction with a thymic cyst, was rendered. Pleural fluid was positive for Hodgkin lymphoma, and a bone marrow biopsy showed no evidence of lymphoma. 

The patient was staged at IIAE and started on chemotherapy with the following regimen: Nitrogen mustard, Prednisone, Vincristine, and Procarbazine (MOPP). He received six cycles of chemotherapy. Chest and mediastinal radiotherapy was initiated given his bulky disease. He received 2 340 cGy to an AP/PA mantle field in 13 fractions with 6 MV photons. 

**Figure 3:**
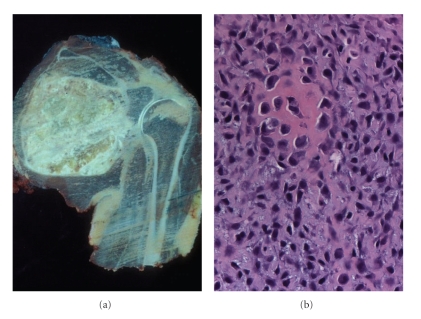
Osteosarcoma: (a) gross photograph of resected specimen demonstrating a large lesion in scapula extending into soft tissue. (b) Representative microscopic section showing pleomorphic and spindle cells with hyperchromatic nuclei and osteoid production. (H&E. 200×).

At the age of 18 years (in the year 2002) he developed a left scapular pain and was clinically presumed to be caused by bursitis. Having the pain persisted for four months, an MRI was ordered disclosing a 10 cm soft tissue mass surrounding the left scapula, which had multiple calcifications and had eroded into the scapula. Chest X-ray showed a single small nodule within the right lower lobe of the lung. Biopsy of the periscapular mass demonstrated a chondroblastic osteosarcoma (Figure 3). The osteosarcoma was present within the field of radiation therapy directed to the chest and mediastinum that had been given following his diagnosis of Hodgkin lymphoma ten years prior to his current presentation. He received Cisplatin, Doxorubicin, and Methotrexate. Subsequently, he had an amputation of the left shoulder and resection of the lung nodule. 

At the age of 22 (in 2006) he presented with fever, cough, night sweats, fatigue, decreased appetite, and a weight loss of 25 pounds. A peripheral blood count showed white blood cells: 6.8 K/cu mm, red blood cells: 2.78 M/cu mm, Hgb: 9.5 g/dL, and platelet: 172,000 K/cu mm. Peripheral blood differential count showed: 3% myelocytes, 2% metamyelocytes, 22% bands, 9% segmented neutrophils, 24% lymphocytes, 38% monocytes, and 2% eosinophils. 

**Figure 4:**
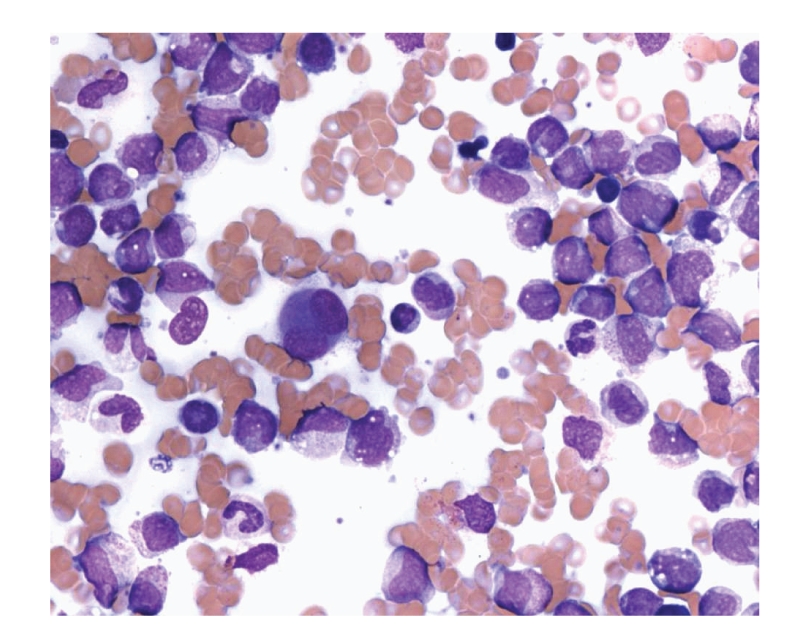
Bone marrow aspirate smear showing medium-to-large sized myeloblasts with moderate cytoplasm, round to lobulated or bean-shaped nuclei, and variably conspicuous nucleoli. (H&E. 400×).

A bone marrow aspirate smear showed increased cellular elements with blasts, constituting 82% of marrow cells. These blasts were medium-to-large sized with scanty-to-moderate cytoplasm, mostly fine red cytoplasmic granules, round to lobulated or bean-shaped nuclei, and variably conspicuous nucleoli (Figure 4). No Auer rods were encountered. Myeloid, erythroid cells, and megakaryocytes were markedly decreased. A differential count on bone marrow aspirate smear showed the following: 10% myeloid cells, 1% erythroid cells, 5% lymphocytes, 2% monocytes, and 82% myeloblasts. Immunophenotyping by flow cytometry showed blasts positivity for CD13, CD33, and myeloperoxidase, CD11b, CD11c, and HLA: DR. A bone marrow biopsy showed an increased cellularity (95%) with replacement of architecture by sheets of myeloblasts. The diagnosis of Secondary AML was made. 

The patient was put on an acute myelogenous leukemia protocol and underwent bone marrow transplantation afterwards. His course was complicated by an EBV-associated lymphoproliferative disease and graft-versus-host disease. His status deteriorated and he developed fever of unknown origin with an altered mental status and tachypnea and he passed away within days of onset of the fever at the age of 23 years.

## 3. Discussion

Mediastinal enlargement in patients with Hodgkin lymphoma (HL) can be attributable to mediastinal lymphadenopathy, thymic involvement by HL, or rarely the presence of thymic cysts [1]. In 1983, Smith et al. reported a case whereby the initial presentation in a 14-year-old boy was that of a large thymic cyst with a microscopic focus of Hodgkin lymphoma discovered in the wall of the cyst [2]. Our case had a similar presentation. 

In 1987, a review of literature reported 11 cases ranging in age between 17 and 40 years. In this series, 60% of the cases were of nodular sclerosis subtype and 40% were of mixed cellularity subtype [1]. There is no documented sex predilection. 

The pathogenesis of cyst formation in the thymus in conjunction of HL is unclear. Possible explanations include a secondary treatment effect or thymic tissue infiltration by neoplastic cells [3]. 

Thymic cysts can be microscopic or macroscopic and can reach sizes of 9–12 cm. They contain clear straw color or hemorrhagic fluid. Microscopically, thymic cysts are lined by thymic epithelial cells with intervening thymic tissue [4]. In cases where there is concurrent lymphoma within the thymus, the classic histology of the Hodgkin subtype is noted. 

The presence of a thymic cyst in patients with Hodgkin lymphoma does not necessitate any specific additional treatment. However, awareness of this association is of importance in raising the differential of a coexisting Hodgkin lymphoma in patients who present with thymic cysts. It also carries implications of posttreatment residual disease. The persistence of the thymic cysts after treatment can confound a completely resolved HL and give the impression of a residual disease [4]. Some authors advocate follow-up of these cysts without any diagnostic or therapeutic intervention based on their experience with no progression of these cysts after 2–6 years of follow-up, although, others suggest that biopsy confirmation of HL is needed before treating such residual thymic cysts [3]. With the increased cure rate in patients with Hodgkin Lymphoma, the risk of secondary malignancy is increased. The relative rate of developing a secondary malignancy in HL survivors is 2.22% [5]. Earlier reports implicated leukemia as the most common secondary malignancy in patients with HL. Recent data showed that 76% of secondary malignancies in HL survivors are solid tumors which may appear in the areas of the lung, breast, bone, thyroid, and soft tissue sarcomas [5]. They occur after a minimal latency of 5–10 years. A number of etiological factors have been suggested and include cancer therapy (chemotherapy and radiotherapy), underlying cancer predisposing syndromes (e.g., Bloom syndrome), or other modifiable factors (smoking, alcohol, and diet) [5]. 

The majority of secondary AML (sAML) occurs in the first year after treatment of the primary malignancy [6]. The risk of developing secondary AML is associated with a number of factors including type of chemotherapeutic agents used, radiotherapy, and some host genetic factors. sAML associated with alkylating agents occurs 5–7 years following therapy and has been shown to be dose-related [7]. Most of these cases are of M1 or M2 subtype. On the other hand, sAML occurring in patients receiving topoisomerase II inhibitors is dose-independent and of myelomonocytic subtype. Prognosis in cases of s-AML is worse than patients with primary AML [7]. 

In a review of cancer registries in a number of countries from 1970 to 2001, Schonfeld et al. noted that the incidence of acute myelogenous leukemia in patient with HL has declined. They propose that this change is probably related to change in chemotherapy [8]. Earlier chemotherapeutic regimens for the treatment of HL used MOPP which had a great efficacy in combating the disease but was associated with toxicity and long-term risk of developing acute leukemia or myelodysplastic syndromes. More recent modalities utilize ABVD which has a lower risk of developing hematopoietic malignancies [9]. 

The National institute of Cancer Childhood Cancer Survivors Study (CCSS) looked at the incidence of secondary malignancies in childhood cancer survivors treated by radiotherapy between 1970 and 1986 [10]. The study subjects were survivors of CNS tumors, Leukemia, HL, non-HL, kidney tumors, neuroblastoma, soft tissue sarcomas, and bone tumors. They found that the risk of subsequent cancer using multivariate analysis was increased in females, childhood cancer of younger age, patients with an initial diagnosis of HL or a soft tissue sarcoma, and in patients treated with an alkylating agent. The main three secondary malignancies in this cohort were tumors of bone (19.14%), breast (16.18%), and thyroid (11.34%) [10]. 

In conclusion, we presented a case of thymic Hodgkin lymphoma in a 9-year-old boy who developed multiple secondary malignancies over a course of 14 years of follow-up. Modifications in therapeutic agents and avoiding radiation therapy may reduce the risks of such complications in the future.
